# Supplementary material for: Patterns of physical activity over time in older patients rehabilitating after hip fracture surgery: a preliminary observational study
Source: BMC Geriatr. 2023 Jun 16;23:373. doi: 10.1186/s12877-023-04054-2 (PMC10276437; doi:10.1186/s12877-023-04054-2)
Supplement: Supplementary file 1 — Additional file 1. Workflow data-analysis raw MOX data. [file 12877_2023_4054_MOESM1_ESM.docx]

Additional File 1. Workflow data-analysis raw MOX data

| Steps for analysing raw MOX data | | Remarks |
| --- | --- | --- |
| 1. Pre-processing | 1. Select data between 7.00 am and 10 pm 2. Detection of missing data:    1. More than 10 missing minutes in one hour? Yes: define hour as missing hour    2. More than 3 missing hours in one day? Yes: define day as a missing day 3. For the remaining days:    1. Moving average filter with a 0.12 second window size    2. High pass filter with a cut-off frequency of 1 Hz    3. Data segmentation with a window size of 2 seconds. | Missing data is detected in the raw data by checking for missing timestamps.  A missing day was classified with a NaN value for the intensity of physical activity.  Removal of noise acceleration and gravitational acceleration using a moving average filter and high pass filter (1).  Data segmentation results in 27,000 windows per day. |
| 2. Calculate the physical activity parameter for each physical activity aspect | 1. Intensity of physical activity:    1. Calculate the Signal Magnitude Area (SMA) for each window of 2 seconds.    2. Take the sum of the SMA of all windows in a day to calculate the overall intensity of physical activity per day (counts/day) 2. Variability in the intensity of physical activity:    1. Data segmentation with a window size of 5 days    2. Calculate the variability for each window with the *“var”* function in Matlab 3. Variability in the intensity of physical activity within each day:    1. Take the SMA of all windows in a day (27.000 windows)    2. Calculate the variability for each day (over all 27.000 windows) with the *“var”* function in Matlab | For the variability in the intensity of physical activity an overlapping, centred sliding window method was used. |
| 1. Plot figures | 1. Smooth the data with a Gaussian-weighted moving average smoothing filter with a window length of 14 days using the function *“smoothdata”* in Matlab 2. Plot the overall intensity of physical activity and the day-to-day variability over time 3. Plot the variability in overall intensity of physical activity for each window | The smoothing filter used an overlapping centred sliding window method. |

1. Bijnens W, Aarts J, Stevens A, Ummels D, Meijer K. Optimization and validation of an adjustable activity classification algorithm for assessment of physical behavior in elderly. Sensors. 2019;19(24):5344.
