# Supplementary material for: Patterns of physical activity over time in older patients rehabilitating after hip fracture surgery: a preliminary observational study
Source: BMC Geriatr. 2023 Jun 16;23:373. doi: 10.1186/s12877-023-04054-2 (PMC10276437; doi:10.1186/s12877-023-04054-2)
Supplement: Supplementary file 2 — Additional file 2. Ratings of all raters for each patient and for each aspect of physical activity. [file 12877_2023_4054_MOESM2_ESM.docx]

Additional file 2. Ratings of all raters for each patient and for each aspect of physical activity

A. Pattern of overall physical activity: ratings of all raters for each patient

| **Enrolled patients** | **Upward linear pattern (n)** | **Hill shape pattern (n)** | **S-shape pattern (n)** | **Flat line pattern (n)** | **Cubic curve pattern**  **(n)** | **Downward linear pattern (n)** | **Else (n)** |
| --- | --- | --- | --- | --- | --- | --- | --- |
| 1 | 0 | 3 | 13 | 0 | 2 | 0 | 0 |
| 2 | 0 | 0 | 1 | 2 | 0 | 1 | 14 |
| 3 | 0 | 0 | 3 | 0 | 15 | 0 | 0 |
| 4 | 0 | 3 | 1 | 2 | 0 | 0 | 12 |
| 5 | 1 | 0 | 5 | 0 | 2 | 0 | 10 |
| 6 | 3 | 11 | 4 | 0 | 0 | 0 | 0 |
| 7 | 0 | 0 | 1 | 0 | 15 | 0 | 2 |
| 8 | 5 | 1 | 7 | 0 | 4 | 0 | 1 |
| 9 | 2 | 6 | 10 | 0 | 0 | 0 | 0 |
| 10 | 0 | 1 | 2 | 0 | 15 | 0 | 0 |
| 11 | 1 | 0 | 2 | 1 | 4 | 0 | 10 |
| 12 | 18 | 0 | 0 | 0 | 0 | 0 | 0 |
| 13 | 1 | 1 | 6 | 0 | 2 | 0 | 8 |
| 14 | 0 | 0 | 0 | 4 | 0 | 3 | 11 |
| 15 | 1 | 0 | 0 | 6 | 0 | 0 | 11 |
| 16 | 15 | 1 | 2 | 0 | 0 | 0 | 0 |
| 17 | 0 | 1 | 0 | 2 | 0 | 0 | 15 |
| 18 | 3 | 0 | 2 | 0 | 13 | 0 | 0 |
| 19 | 0 | 0 | 14 | 0 | 3 | 0 | 1 |
| 20 | 0 | 0 | 0 | 1 | 6 | 0 | 11 |
| 21 | 4 | 0 | 1 | 1 | 5 | 0 | 7 |
| 22 | 2 | 0 | 12 | 0 | 2 | 0 | 2 |
| 23 | 4 | 2 | 12 | 0 | 0 | 0 | 0 |
| 24 | 18 | 0 | 0 | 0 | 0 | 0 | 0 |
| 25 | 18 | 0 | 0 | 0 | 0 | 0 | 0 |
| 26 | 0 | 1 | 17 | 0 | 0 | 0 | 0 |
| 27 | 10 | 0 | 6 | 0 | 2 | 0 | 0 |
| 28 | 18 | 0 | 0 | 0 | 0 | 0 | 0 |
| 29 | 12 | 5 | 1 | 0 | 0 | 0 | 0 |
| 30 | 0 | 0 | 0 | 2 | 3 | 0 | 13 |
| 31 | 7 | 0 | 5 | 0 | 1 | 0 | 5 |
| 32 | 0 | 0 | 18 | 0 | 0 | 0 | 0 |
| 33 | 1 | 2 | 8 | 0 | 4 | 0 | 3 |
| 34 | 17 | 0 | 1 | 0 | 0 | 0 | 0 |
| 35 | 0 | 5 | 10 | 0 | 0 | 0 | 3 |
| 36 | 1 | 0 | 16 | 0 | 0 | 0 | 1 |
| 37 | 0 | 1 | 17 | 0 | 0 | 0 | 0 |
| 38 | 1 | 2 | 15 | 0 | 0 | 0 | 0 |
| 39 | 1 | 2 | 3 | 0 | 3 | 0 | 9 |
| 40 | 0 | 1 | 4 | 0 | 4 | 0 | 9 |
| 41 | 3 | 13 | 2 | 0 | 0 | 0 | 0 |
| 42 | 4 | 1 | 2 | 0 | 11 | 0 | 0 |
| 43 | 7 | 0 | 6 | 0 | 2 | 0 | 3 |
| 44 | 0 | 0 | 13 | 0 | 1 | 0 | 4 |
| 45 | 0 | 0 | 0 | 6 | 0 | 0 | 12 |
| 46 | 0 | 1 | 17 | 0 | 0 | 0 | 0 |
| 47 | 1 | 0 | 15 | 0 | 0 | 0 | 2 |
| 48 | 0 | 0 | 13 | 0 | 1 | 0 | 4 |
| 49 | 16 | 1 | 1 | 0 | 0 | 0 | 0 |
| 50 | 16 | 1 | 0 | 1 | 0 | 0 | 0 |
| 51 | 16 | 0 | 2 | 0 | 0 | 0 | 0 |
| 52 | 2 | 7 | 9 | 0 | 0 | 0 | 0 |
| 53 | 0 | 0 | 15 | 0 | 0 | 0 | 3 |
| 54 | 0 | 1 | 0 | 2 | 2 | 1 | 12 |
| 55 | 4 | 1 | 7 | 0 | 3 | 0 | 3 |
| 56 | 1 | 0 | 15 | 0 | 0 | 0 | 2 |
| 57 | 0 | 0 | 16 | 0 | 1 | 0 | 1 |
| 58 | 5 | 11 | 1 | 0 | 1 | 0 | 0 |
| 59 | 0 | 6 | 0 | 0 | 7 | 0 | 5 |
| 60 | 17 | 0 | 1 | 0 | 0 | 0 | 0 |
| 61 | 3 | 15 | 0 | 0 | 0 | 0 | 0 |
| 62 | 0 | 17 | 0 | 0 | 0 | 0 | 1 |
| 63 | 2 | 0 | 0 | 0 | 15 | 0 | 1 |
| 64 | 0 | 4 | 0 | 1 | 0 | 1 | 12 |
| 65 | 0 | 1 | 1 | 0 | 5 | 0 | 11 |
| 66 | 0 | 2 | 16 | 0 | 0 | 0 | 0 |

B. Pattern of variability in overall physical activity: ratings of all raters for each patient

| **Enrolled patients** | **Wave pattern (n)** | **N-shape pattern (n)** | **S-shape pattern (n)** | **Bell shape pattern (n)** | | **Mountain pattern**  **(n)** | **Reverse s-shape pattern (n)** | **Else (n)** |
| --- | --- | --- | --- | --- | --- | --- | --- | --- |
| 1 | 0 | 6 | 1 | 1 | 5 | | 0 | 5 |
| 2 | 0 | 0 | 0 | 17 | 0 | | 0 | 1 |
| 3 | 2 | 0 | 11 | 0 | 0 | | 0 | 5 |
| 4 | 0 | 0 | 0 | 18 | 0 | | 0 | 0 |
| 5 | 1 | 0 | 0 | 8 | 4 | | 0 | 5 |
| 6 | 8 | 1 | 6 | 1 | 0 | | 0 | 2 |
| 7 | 0 | 0 | 0 | 0 | 11 | | 0 | 7 |
| 8 | 0 | 0 | 0 | 18 | 0 | | 0 | 0 |
| 9 | 0 | 0 | 0 | 15 | 0 | | 0 | 3 |
| 10 | 0 | 0 | 0 | 0 | 0 | | 12 | 6 |
| 11 | 13 | 0 | 3 | 0 | 0 | | 0 | 2 |
| 12 | 0 | 0 | 17 | 0 | 0 | | 0 | 1 |
| 13 | 1 | 1 | 1 | 6 | 0 | | 0 | 9 |
| 14 | 0 | 7 | 0 | 6 | 4 | | 0 | 1 |
| 15 | 2 | 1 | 0 | 0 | 1 | | 0 | 14 |
| 16 | 0 | 0 | 0 | 14 | 0 | | 2 | 2 |
| 17 | 3 | 0 | 1 | 0 | 0 | | 0 | 14 |
| 18 | 17 | 1 | 0 | 0 | 0 | | 0 | 0 |
| 19 | 5 | 2 | 6 | 1 | 0 | | 0 | 4 |
| 20 | 0 | 3 | 0 | 5 | 2 | | 0 | 8 |
| 21 | 0 | 2 | 0 | 0 | 11 | | 0 | 5 |
| 22 | 1 | 0 | 15 | 0 | 0 | | 0 | 2 |
| 23 | 0 | 0 | 0 | 0 | 14 | | 0 | 4 |
| 24 | 7 | 0 | 9 | 0 | 0 | | 0 | 2 |
| 25 | 0 | 0 | 18 | 0 | 0 | | 0 | 0 |
| 26 | 7 | 1 | 7 | 0 | 0 | | 0 | 3 |
| 27 | 0 | 0 | 15 | 0 | 0 | | 0 | 3 |
| 28 | 0 | 0 | 16 | 0 | 0 | | 0 | 2 |
| 29 | 5 | 3 | 0 | 0 | 3 | | 0 | 7 |
| 30 | 1 | 0 | 0 | 0 | 1 | | 0 | 16 |
| 31 | 3 | 0 | 8 | 0 | 0 | | 0 | 7 |
| 32 | 15 | 3 | 0 | 0 | 0 | | 0 | 0 |
| 33 | 8 | 7 | 0 | 0 | 0 | | 0 | 3 |
| 34 | 0 | 18 | 0 | 0 | 0 | | 0 | 0 |
| 35 | 11 | 5 | 0 | 0 | 0 | | 0 | 2 |
| 36 | 1 | 3 | 0 | 0 | 9 | | 0 | 5 |
| 37 | 0 | 13 | 0 | 2 | 0 | | 0 | 3 |
| 38 | 0 | 0 | 0 | 18 | 0 | | 0 | 0 |
| 39 | 0 | 6 | 0 | 0 | 4 | | 0 | 8 |
| 40 | 0 | 0 | 0 | 0 | 18 | | 0 | 0 |
| 41 | 0 | 0 | 0 | 0 | 0 | | 12 | 6 |
| 42 | 11 | 1 | 2 | 0 | 0 | | 0 | 4 |
| 43 | 0 | 10 | 0 | 1 | 5 | | 0 | 2 |
| 44 | 0 | 10 | 1 | 2 | 0 | | 0 | 5 |
| 45 | 4 | 5 | 0 | 0 | 1 | | 0 | 8 |
| 46 | 0 | 0 | 0 | 5 | 11 | | 0 | 2 |
| 47 | 1 | 9 | 0 | 6 | 0 | | 0 | 2 |
| 48 | 0 | 0 | 0 | 0 | 11 | | 0 | 7 |
| 49 | 0 | 18 | 0 | 0 | 0 | | 0 | 0 |
| 50 | 0 | 0 | 0 | 0 | 0 | | 13 | 5 |
| 51 | 0 | 0 | 9 | 6 | 0 | | 0 | 3 |
| 52 | 0 | 12 | 0 | 3 | 1 | | 0 | 2 |
| 53 | 0 | 0 | 2 | 15 | 0 | | 0 | 1 |
| 54 | 1 | 1 | 0 | 0 | 10 | | 0 | 6 |
| 55 | 1 | 16 | 0 | 0 | 1 | | 0 | 0 |
| 56 | 0 | 0 | 0 | 18 | 0 | | 0 | 0 |
| 57 | 0 | 9 | 2 | 0 | 3 | | 0 | 4 |
| 58 | 17 | 0 | 1 | 0 | 0 | | 0 | 0 |
| 59 | 2 | 1 | 0 | 0 | 4 | | 0 | 11 |
| 60 | 0 | 0 | 14 | 0 | 0 | | 0 | 4 |
| 61 | 0 | 0 | 0 | 0 | 0 | | 17 | 1 |
| 62 | 14 | 0 | 0 | 0 | 0 | | 0 | 4 |
| 63 | 5 | 8 | 0 | 1 | 0 | | 0 | 4 |
| 64 | 0 | 0 | 0 | 0 | 0 | | 18 | 0 |
| 65 | 0 | 0 | 0 | 0 | 11 | | 0 | 7 |
| 66 | 2 | 16 | 0 | 0 | 0 | | 0 | 0 |

C. Pattern of day-to-day variability: ratings of all raters for each patient

| **Enrolled patients** | **Upward linear pattern (n)** | **Hill pattern (n)** | **S-shape pattern (n)** | **Bell shape pattern (n)** | **Cubic curve pattern (n)** | **Else (n)** |
| --- | --- | --- | --- | --- | --- | --- |
| 1 | 0 | 5 | 1 | 0 | 4 | 8 |
| 2 | 0 | 0 | 0 | 11 | 0 | 7 |
| 3 | 4 | 0 | 2 | 0 | 11 | 1 |
| 4 | 0 | 0 | 0 | 14 | 0 | 4 |
| 5 | 0 | 0 | 1 | 0 | 1 | 16 |
| 6 | 1 | 4 | 12 | 0 | 1 | 0 |
| 7 | 0 | 0 | 1 | 0 | 9 | 8 |
| 8 | 14 | 0 | 2 | 0 | 0 | 2 |
| 9 | 0 | 6 | 12 | 0 | 0 | 0 |
| 10 | 0 | 1 | 2 | 0 | 14 | 1 |
| 11 | 0 | 0 | 1 | 10 | 1 | 6 |
| 12 | 16 | 2 | 0 | 0 | 0 | 0 |
| 13 | 0 | 0 | 13 | 0 | 1 | 4 |
| 14 | 0 | 0 | 0 | 4 | 1 | 13 |
| 15 | 0 | 1 | 6 | 2 | 0 | 9 |
| 16 | 5 | 2 | 11 | 0 | 0 | 0 |
| 17 | 0 | 0 | 0 | 1 | 3 | 14 |
| 18 | 0 | 0 | 1 | 0 | 10 | 7 |
| 19 | 0 | 0 | 11 | 1 | 3 | 3 |
| 20 | 0 | 0 | 0 | 0 | 8 | 10 |
| 21 | 0 | 0 | 6 | 0 | 3 | 9 |
| 22 | 0 | 0 | 11 | 0 | 2 | 5 |
| 23 | 0 | 1 | 14 | 1 | 1 | 1 |
| 24 | 18 | 0 | 0 | 0 | 0 | 0 |
| 25 | 18 | 0 | 0 | 0 | 0 | 0 |
| 26 | 0 | 0 | 17 | 1 | 0 | 0 |
| 27 | 1 | 1 | 15 | 0 | 0 | 1 |
| 28 | 18 | 0 | 0 | 0 | 0 | 0 |
| 29 | 5 | 8 | 5 | 0 | 0 | 0 |
| 30 | 0 | 0 | 0 | 3 | 2 | 13 |
| 31 | 16 | 0 | 1 | 0 | 1 | 0 |
| 32 | 2 | 1 | 15 | 0 | 0 | 0 |
| 33 | 0 | 0 | 9 | 0 | 4 | 5 |
| 34 | 0 | 1 | 1 | 0 | 11 | 5 |
| 35 | 0 | 3 | 2 | 8 | 0 | 5 |
| 36 | 1 | 0 | 12 | 0 | 2 | 3 |
| 37 | 0 | 1 | 7 | 9 | 0 | 1 |
| 38 | 0 | 2 | 14 | 1 | 0 | 1 |
| 39 | 0 | 0 | 2 | 8 | 0 | 8 |
| 40 | 0 | 0 | 6 | 0 | 3 | 9 |
| 41 | 0 | 15 | 2 | 0 | 0 | 1 |
| 42 | 3 | 0 | 3 | 0 | 9 | 3 |
| 43 | 0 | 1 | 7 | 0 | 6 | 4 |
| 44 | 0 | 0 | 11 | 0 | 1 | 6 |
| 45 | 0 | 0 | 0 | 5 | 0 | 13 |
| 46 | 0 | 1 | 17 | 0 | 0 | 0 |
| 47 | 0 | 1 | 15 | 2 | 0 | 0 |
| 48 | 0 | 0 | 15 | 2 | 0 | 1 |
| 49 | 9 | 4 | 5 | 0 | 0 | 0 |
| 50 | 4 | 14 | 0 | 0 | 0 | 0 |
| 51 | 10 | 0 | 7 | 0 | 0 | 1 |
| 52 | 0 | 2 | 16 | 0 | 0 | 0 |
| 53 | 0 | 0 | 11 | 0 | 2 | 5 |
| 54 | 0 | 0 | 0 | 8 | 1 | 9 |
| 55 | 0 | 0 | 0 | 0 | 4 | 14 |
| 56 | 0 | 1 | 16 | 0 | 0 | 1 |
| 57 | 0 | 1 | 15 | 0 | 0 | 2 |
| 58 | 0 | 13 | 5 | 0 | 0 | 0 |
| 59 | 0 | 3 | 1 | 0 | 7 | 7 |
| 60 | 15 | 0 | 1 | 0 | 1 | 1 |
| 61 | 1 | 17 | 0 | 0 | 0 | 0 |
| 62 | 0 | 16 | 2 | 0 | 0 | 0 |
| 63 | 0 | 1 | 1 | 0 | 12 | 4 |
| 64 | 0 | 1 | 0 | 17 | 0 | 0 |
| 65 | 0 | 1 | 0 | 6 | 1 | 10 |
| 66 | 0 | 1 | 17 | 0 | 0 | 0 |
