# Supplementary material for: Patterns of physical activity over time in older patients rehabilitating after hip fracture surgery: a preliminary observational study
Source: BMC Geriatr. 2023 Jun 16;23:373. doi: 10.1186/s12877-023-04054-2 (PMC10276437; doi:10.1186/s12877-023-04054-2)
Supplement: Supplementary file 3 — Additional file 3. Patient characteristics for each pattern of physical activity of all three aspects of physical activity. [file 12877_2023_4054_MOESM3_ESM.docx]

Additional file 3. Patient characteristics for each pattern of physical activity of all three aspects of physical activity

A. Pattern of overall physical activity

|  | **Total**  **(n=66)** | **Upward linear pattern (n=15)** | **Hill shape pattern (n=6)** | **S-shape pattern (n=23)** | **Cubic curve pattern (n=6)** | **Else**  **(n=16)** | **p-value** |
| --- | --- | --- | --- | --- | --- | --- | --- |
| **Age**; median (IQR) | 83 (79-88) | 82 (81-87) | 84 (79-89) | 83 (80-90) | 83 (78-84) | 83 (78-88) | 0.90 |
| **Female gender**; n (%) | 49 (74.2) | 12 (80) | 4 (66.7) | 15 (65.2) | 5 (83.3) | 13 (81.3) | 0.79 |
| **Premorbid living situation**; n (%)  Home  Home with care  Residential home  Nursing home | 47 (71.2)  17 (25.8)  1 (1.5)  1 (1.5) | 10 (66.6)  4 (26.7)  1 (6.7)  - | 5 (83.3)  1 (16.7)  -  - | 16 (69.6)  7 (30.4)  -  - | 6 (100)  -  -  - | 10 (62.5)  5 (31.3)  -  1 (6.3) | 0.64 |
| **PFMS**; n (%)  1  2  3  4  5 | 27 (40.9)  6 (9.1)  33 (50)  -  - | 9 (60)  1 (6.7)  5 (33.3)  -  - | 2 (33.3)  -  4 (66.7)  -  - | 10 (43.5)  2 (8.7)  11 (47.8)  -  - | 2 (33.3)  1 (16.7)  3 (50)  -  - | 4 (25)  2 (12.5)  10 (62.5)  -  - | 0.69 |
| **Premorbid Katz-ADL**; median (IQR) | 0 (0-1) | 0 (0-0) | 0 (0-0) | 0 (0-1) | 0 (0-0) | 0 (0-1) | 0.17 |
| **Charlson Comorbidity Index**; median (IQR) | 1 (0-2) | 0 (0-3) | 1 (0-1) | 1 (0-2) | 0 (0-1) | 1 (1-2) | 0.37 |
| **Surgical treatment**; n (%)  Hemiarthroplasty  Intramedullary implant  Dynamic Hip Screw | 25 (37.9)  36 (54.5)  5 (7.6) | 6 (40)  7 (46.7)  2 (13.3) | 3 (50)  3 (50)  - | 8 (34.8)  13 (56.5)  2 (8.7) | 1 (16.7)  4 (66.7)  1 (16.7) | 7 (43.8)  9 (56.3)  - | 0.80 |
| **Weight bearing protocol**; n (%)  Non weight bearing  Partial weight bearing  Full weight bearing | -  3 (4.5)  63 (95.5) | -  -  15 (100) | -  -  6 (100) | -  2 (8.7)  21 (91.3) | -  1 (16.7)  5 (83.3) | -  -  16 (100) | 0.29 |
| **MoCA**; median (IQR) ^a^ | 21 (17-24) | 22 (20-25) | 20 (18-21) | 21 (17-25) | 23 (21-25) | 17 (16-21) | 0.07 |
| **BI admission rehabilitation**; median (IQR) ^b^ | 12 (9-14) | 15 (11-16) | 13 (12-16) | 11 (9-12) | 9 (9-11) | 9 (8-13) | **0.03** |
| **FMS admission rehabilitation**; n (%)  1  2  3  4  5 | -  -  7 (10.6)  55 (83.3)  4 (6.1) | -  -  5 (33.3)  10 (66.7)  - | -  -  -  6 (100)  - | -  -  -  21 (91.3)  2 (8.7) | -  -  -  5 (83.3)  1 (16.7) | -  -  2 (12.5)  13 (81.3)  1 (6.3) | 0.06 |
| **FAC score admission rehab**; n (%)  0  1  2  3  4  5 | 4 (6.1)  4 (6.1)  18 (27.3)  30 (45.4)  10 (15.1)  - | -  -  1 (6.7)  8 (53.5)  6 (40)  - | -  -  1 (16.7)  3 (50)  2 (33.3)  - | 2 (8.7)  2 (8.7)  7 (30.4)  11 (47.8)  1 (4.3)  - | 1 (16.7)  -  3 (50)  1 (16.7)  1 (16.7)  - | 1 (6.3)  2 (12.5)  6 (37.5)  7 (43.8)  -  - | 0.05 |
| **Complications during rehabilitation**; n (%)  Yes  No | 13 (19.7)  53 (80.3) | 1 (6.7)  14 (93.3) | 2 (33.3)  4 (66.7) | 2 (8.7)  21 (91.3) | 2 (33.3)  4 (66.7) | 6 (37.5)  10 (62.5) | 0.07 |
| **Duration of rehabilitation stay**; median (IQR) | 28 (21-42) | 16 (12-24) | 21 (10-30) | 30 (23-41) | 42 (21-47) | 42 (31-52) | **<0.001** |
| **BI discharge rehabilitation**; median (IQR) ^c^ | 16 (15-18) | 17 (17-18) | 17 (16-18) | 16 (15-18) | 17 (15-18) | 16 (14-17) | 0.21 |
| **FMS discharge rehabilitation**; n (%) ^d^  1  2  3  4  5 | -  7 (10.9)  42 (65.6)  14 (21.9)  1 (1.5) | -  3 (20)  11 (73.3)  1 (6.7)  - | -  -  5 (83.3)  1 (16.7)  - | -  3 (13.6)  13 (59.1)  6 (27.3)  - | -  1 (16.7)  5 (83.3)  -  - | -  -  8 (53.3)  6 (40)  1 (6.7) | 0.24 |
| **FAC score discharge rehabilitation**; n (%)  0  1  2  3  4  5 | -  -  2 (3)  2 (3)  51 (77.3)  11 (16.7) | -  -  -  -  10 (66.7)  5 (33.3) | -  -  -  -  5 (83.3)  1 (16.7) | -  -  -  -  21 (91.3)  2 (8.7) | -  -  -  -  5 (83.3)  1 (16.7) | -  -  2 (12.5)  2 (12.5)  10 (62.5)  2 (12.5) | 0.19 |
| BI = Barthel Index, (P)FMS = (Pre-)Fracture Mobility Score, MoCA = Montreal Cognitive Assessment, FAC = Functional Ambulation Categories  ^a^ number of missing = 16, ^b^ number of missing = 8, ^c^ number of missing = 3, ^d^ number of missing = 2 | | | | | | | |

B. Pattern of variability in overall physical activity

|  | **Total**  **(n=66)** | **Wave pattern (n=10)** | **N-shape pattern (n=141)** | **S-shape pattern (n=10)** | **Bell shape pattern (n=9)** | **Mountain shape pattern (n=9)** | **Reverse s-shape pattern (n=5)** | **Else**  **(n=9)** | **p-value** |
| --- | --- | --- | --- | --- | --- | --- | --- | --- | --- |
| **Age**; median (IQR) | 83 (79-88) | 83 (82-89) | 84 (82-87) | 81 (74-86) | 84 (79-90) | 82 (75-85) | 83 (83-84) | 80 (79-88) | 0.83 |
| **Female gender**; n (%) | 49 (74.2) | 6 (60) | 13 (92.9) | 9 (90) | 4 (44.4) | 6 (66.7) | 4 (80) | 7 (77.8) | 0.14 |
| **Premorbid living situation**; n (%)  Home  Home with care  Residential home  Nursing home | 47 (71.2)  17 (25.8)  1 (1.5)  1 (1.5) | 8 (80)  2 (20)  -  - | 11 (78.6)  3 (21.4)  -  - | 6 (60)  4 (40)  -  - | 6 (66.7)  3 (33.3)  -  - | 6 (66.7)  3 (33.3)  -  - | 4 (80)  -  1 (20)  - | 6 (66.7)  2 (22.2)  -  1 (11.1) | 0.64 |
| **PFMS**; n (%)  1  2  3  4  5 | 27 (40.9)  6 (9.1)  33 (50)  -  - | 2 (20)  1 (10)  7 (70)  -  - | 7 (50)  -  7 (50)  -  - | 4 (40)  1 (10)  5 (50)  -  - | 4 (44.4)  3 (33.3)  2 (22.3)  -  - | 4 (44.4)  -  5 (55.6)  -  - | 2 (40)  1 (20)  2 (40)  -  - | 4 (44.4)  -  5 (55.6)  -  - | 0.43 |
| **Premorbid Katz-ADL**; median (IQR) | 0 (0-1) | 0 (0-1) | 0 (0-0) | 0 (0-1) | 0 (0-1) | 0 (0-1) | 0 (0-0) | 0 (0-1) | 0.56 |
| **Charlson Comorbidity Index**; median (IQR) | 1 (0-2) | 1 (1-2) | 1 (0-2) | 0 (0-3) | 1 (0-3) | 1 (0-1) | 1 (0-1) | 1 (1-2) | 0.65 |
| **Surgical treatment**; n (%)  Hemiarthroplasty  Intramedullary implant  Dynamic Hip Screw | 25 (37.9)  36 (54.5)  5 (7.6) | 4 (40)  6 (60)  - | 5 (35.7)  9 (64.3)  - | 3 (30)  5 (50)  2 (20) | 6 (66.7)  3 (33.3)  - | 2 (22.2)  6 (66.7)  1 (11.1 | 3 (60)  1 (20)  1 (20) | 2 (22.2)  6 (66.7)  1 (11.1) | 0.36 |
| **Weight bearing protocol**; n (%)  Non weight bearing  Partial weight bearing  Full weight bearing | -  3 (4.5)  63 (95.5) | -  -  10(100) | -  -  14 (100) | -  1 (10)  9 (90) | -  -  9 (100) | -  1 (11.1)  8 (88.9) | -  -  5 (100) | -  1 (11.1)  8 (88.9) | 0.58 |
| **MoCa**; median (IQR) ^a^ | 21 (17-24) | 23 (21-25) | 20 (17-24) | 23 (21-25) | 20 (17-22) | 17 (15-20) | 19 (18-20) | 21 (18-24) | 0.08 |
| **BI admission rehabilitation**; median (IQR) ^b^ | 12 (9-14) | 12 (10-14) | 10 (8-11) | 13 (12-16) | 12 (10-15) | 9 (8-12) | 14 (12-16) | 11 (9-14) | 0.17 |
| **FMS admission rehabilitation**;  1  2  3  4  5 | -  -  7 (10.6)  55 (83.3)  4 (6.1) | -  -  -  10 (100)  - | -  -  -  13 (92.9)  1 (7.1) | -  -  2 (20)  7 (70)  1 (10) | -  -  2 (22.2)  7 (77.8)  - | -  -  1 (11.1)  7 (77.8)  1 (11.1) | -  -  -  5 (100)  - | -  -  2 (22.2)  6 (66.7)  1 (11.1) | 0.49 |
| **FAC score admission rehabilitation**; n (%)  0  1  2  3  4  5 | 4 (6.1)  4 (6.1)  18 (27.3)  30 (45.4)  10 (15.1)  - | -  1 (10)  5 (50)  4 (40)  -  - | 1 (7.1)  -  5 (35.7)  7 (50)  1 (7.1)  - | 1 (10)  -  1 (10)  5 (50)  3 (30)  - | -  -  3 (33.3)  4 (44.5)  2 (22.2)  - | 1 (11.1)  2 (22.2)  3 (33.3)  3 (33.3)  -  - | -  -  -  2 (40)  3 (60)  - | 1 (11.1)  1 (11.1)  1 (11.1)  5 (55.6)  1 (11.1)  - | 0.29 |
| **Complications during rehabilitation**; n (%)  Yes  No | 13 (19.7)  53 (80.3) | -  10 (100) | 3 (21.4)  11 (78.6) | 2 (20)  8 (80) | 3 (33.3)  6 (66.7) | 2 (22.2)  7 (77.8) | 1 (20)  4 (80) | 2 (22.2)  7 (77.8) | 0.67 |
| **Duration of rehabilitation stay**; median (IQR) | 28 (21-42) | 33 (21-42) | 35 (27-48) | 15 (11-25) | 23 (21-29) | 42 (41-65) | 10 (9-17) | 32 (21-42) | **<0.001** |
| **BI discharge rehabilitation**; median (IQR) ^c^ | 16 (15-18) | 16 (15-18) | 16 (15-18) | 18 (16-18) | 14 (14-17) | 17 (16-17) | 17 (16-17) | 16 (14-17) | 0.39 |
| **FMS discharge rehabilitation**; n (%) ^d^  1  2  3  4  5 | -  7 (10.9)  42 (65.6)  14 (21.9)  1 (1.5) | -  1 (10)  7 (70)  2 (20)  - | -  1 (7.7)  7 (53.8)  5 (38.5)  - | -  1 (10)  9 (90)  -  - | -  1 (12.5)  5 (62.5)  1 (12.5)  1 (12.5) | -  2 (22.2)  4 (44.5)  3 (33.3)  - | -  -  4 (80)  1 (20)  - | -  1 (11.1)  6 (67.7)  2 (22.2)  - | 0.70 |
| **FAC score discharge rehab**; n (%)  0  1  2  3  4  5 | -  -  2 (3)  2 (3)  51 (77.3)  11 (16.7) | -  -  -  -  8 (80)  2 (20) | -  -  -  1 (7.1)  11 (78.6)  2 (14.3) | -  -  -  -  8 (80)  2 (20) | -  -  1 (11.1)  -  7 (77.8)  1 (11.1) | -  -  -  -  7 (77.8)  2 (22.2) | -  -  -  -  4 (80)  1 (20) | -  -  1 (11.1)  1 (11.1)  6 (66.7)  1 (11.1) | 0.99 |
| BI = Barthel Index, (P)FMS = (Pre-)Fracture Mobility Score, MoCA = Montreal Cognitive Assessment, FAC = Functional Ambulation Categories  ^a^ number of missing = 16, ^b^ number of missing = 8, ^c^ number of missing = 3, ^d^ number of missing = 2 | | | | | | | | | |

C. Pattern of day-to-day variability

|  | **Total**  **(n = 66)** | **Upward linear pattern (n=9)** | **Hill shape pattern (n=6)** | **S-shape pattern (n=23)** | **Bell shape pattern (n=7)** | **Cubic curve pattern (n=7)** | **Else**  **(n=14)** | **p-value** |
| --- | --- | --- | --- | --- | --- | --- | --- | --- |
| **Age**; median (IQR) | 83 (79-88) | 82 (81-86) | 84 (75-89) | 83 (79-86) | 83 (83-90) | 82 (79-84) | 83 (78-91) | 0.96 |
| **Female gender**; n (%) | 49 (74.2) | 7 (77.8) | 4 (66.7) | 15 (65.2) | 7 (100) | 6 (85.7) | 10 (71.4) | 0.57 |
| **Premorbid living situation**; n (%)  Home  Home with care  Residential home  Nursing home | 47 (71.2)  17 (25.8)  1 (1.5)  1 (1.5) | 6 (66.7)  3 (33.3)  -  - | 5 (83.3)  -  1 (16.7)  - | 15 (65.2)  8 (34.8)  -  - | 4 (57.1)  3 (42.9)  -  - | 7 (100)  -  -  - | 10 (71.4)  3 (21.4)  -  1 (7.1) | 0.21 |
| **PFMS**; n (%)  1  2  3  4  5 | 27 (40.9)  6 (9.1)  33 (50)  -  - | 4 (44.4)  1 (11.2)  4 (44.4)  -  - | 3 (50)  -  3 (50)  -  - | 11 (47.8)  2 (8.7)  10 (43.5)  -  - | 2 (28.6)  2 (28.6)  3 (42.9)  -  - | 2 (28.6)  1 (14.3)  4 (57.1)  -  - | 5 (35.7)  -  9 (64.3)  -  - | 0.78 |
| **Premorbid Katz-ADL**; median (IQR) | 0 (0-1) | 0 (0-0) | 0 (0-0) | 0 (0-1) | 0 (0-1) | 0 (0-0) | 0 (0-0) | 0.47 |
| **Charlson Comorbidity Index**; median (IQR) | 1 (0-2) | 0 (0-0) | 1 (0-1) | 1 (0-2) | 1 (1-3) | 1 (0-2) | 1 (1-2) | 0.42 |
| **Surgical treatment**; n (%)  Hemiarthroplasty  Intramedullary implant  Dynamic Hip Screw | 25 (37.9)  36 (54.5)  5 (7.6) | 4 (44.4)  4 (44.4)  1 (11.2) | 3 (50)  2 (33.3)  1 (16.7) | 8 (34.8)  13 (56.5)  2 (8.7) | 2 (28.6)  5 (71.4)  - | 2 (28.6)  5 (71.4)  - | 6 (42.9)  7 (50)  1 (7.1) | 0.96 |
| **Weight bearing protocol**; n (%)  Non weight bearing  Partial weight bearing  Full weight bearing | -  3 (4.5)  63 (95.5) | -  -  9 (100) | -  -  6 (100) | -  2 (8.7)  21 (91.3) | -  -  7 (100) | -  -  7 (100) | -  1 (7.1)  13 (92.9) | 1.0 |
| **MoCA**; median (IQR) ^a^ | 21 (17-24) | 23 (21-25) | 20 (19-25) | 21 (18-25) | 17 (16-17) | 24 (21-25) | 18 (17-21) | **0.04** |
| **BI admission rehabilitation**; median (IQR) ^b^ | 12 (9-14) | 15 (13-17) | 14 (13-15) | 11 (10-12) | 11 (8-15) | 10 (9-11) | 8 (8-12) | **0.005** |
| **FMS admission rehabilitation**; n (%)  1  2  3  4  5 | -  -  7 (10.6)  55 (83.3)  4 (6.1) | -  -  4 (44.4)  5 (55.6)  - | -  -  -  6 (100)  - | -  -  1 (4.3)  21 (91.3)  1 (4.3) | -  -  -  7 (100)  - | -  -  7 (100)  - | -  -  2 (14.3)  9 (64.3)  3 (21.4) | **0.04** |
| **FAC score admission rehabilitation**; n (%)  0  1  2  3  4  5 | 4 (6.1)  4 (6.1)  18 (27.3)  30 (45.4)  10 (15.1)  - | -  -  -  5 (55.6)  4 (44.4)  - | -  -  1 (16.7)  2 (33.3)  3 (50)  - | 1 (4.3)  2 (8.7)  8 (34.8)  10 (43.5)  2 (8.7)  - | -  -  3 (42.9)  4 (57.1)  -  - | -  -  4 (57.1)  2 (28.6)  1 (14.3)  - | 3 (21.4)  2 (14.3)  2 (14.3)  7 (50)  -  - | 0.06 |
| **Complications during rehabilitation**; n (%)  Yes  No | 13 (19.7)  53 (80.3) | 1 (11.1)  8 (88.9) | 1 (16.7)  5 (83.3) | 3 (13)  20 (87) | 1 (14.3)  6 (85.7) | 2 (28.6)  5 (71.4) | 5 (35.7)  9 (64.3) | 0.60 |
| **Duration of rehabilitation stay**; median (IQR) | 28 (21-42) | 15 (11-22) | 14 (9-21) | 30 (23-41) | 29 (23-42) | 32 (24-42) | 42 (32-65) | **<0.001** |
| **BI discharge rehabilitation**; median (IQR) ^c^ | 16 (15-18) | 17 (15-18) | 17 (16-17) | 17 (16-18) | 16 (15-18) | 16 (16-17) | 16 (13-17) | 0.37 |
| **FMS discharge rehabilitation**; n (%) ^d^  1  2  3  4  5 | -  7 (10.9)  42 (65.6)  14 (21.9)  1 (1.5) | -  1 (11.1)  7 (77.8)  1 (11.1)  - | -  -  5 (83.3)  1 (16.7)  - | -  5 (22.7)  12 (54.5)  5 (27.7)  - | -  -  4 (66.7)  1 (16.7)  1 (16.7) | -  -  7 (100)  -  - | -  1 (7.1)  7 (50)  6 (42.9)  - | 0.28 |
| **FAC score discharge rehab**; n (%)  0  1  2  3  4  5 | -  -  2 (3)  2 (3)  51 (77.3)  11 (16.7) | -  -  -  -  6 (66.7)  3 (33.3) | -  -  -  -  5 (83.3)  1 (16.7) | -  -  -  -  19 (82.6)  4 (17.4) | -  -  1 (14.3)  -  5 (71.4)  1 (14.3) | -  -  -  -  7 (100)  - | -  -  1 (7.2)  2 (14.3)  9 (64.3)  2 (14.3) | 0.47 |
| BI = Barthel Index, (P)FMS = (Pre-)Fracture Mobility Score, MoCA = Montreal Cognitive Assessment, FAC = Functional Ambulation Categories  ^a^ number of missing = 16, ^b^ number of missing = 8, ^c^ number of missing = 3, ^d^ number of missing = 2 | | | | | | | | |
